# Health systems changes after decentralisation: progress, challenges and dynamics in Pakistan

**DOI:** 10.1136/bmjgh-2018-001013

**Published:** 2019-01-30

**Authors:** Shehla Abbas Zaidi, Maryam Bigdeli, Etienne V Langlois, Atif Riaz, David W Orr, Nasir Idrees, Jesse B Bump

**Affiliations:** 1 Department of Community Health Sciences & Women & Child Health Division, Aga Khan University, Karachi, Pakistan; 2 World Health Organization, Geneva, Switzerland; 3 Alliance for Health Policy and Systems Research, World Health Organization, Geneva, Switzerland; 4 Department of Politics and International Studies, University of Cambridge, Cambridge, UK; 5 Independent Governance Consultant, Islamabad, Pakistan; 6 Harvard T.H. Chan School of Public Health, Boston, Massachusetts, USA

**Keywords:** decentralization, health systems, politics, Pakistan

## Abstract

Decentralisation is widely practised but its scrutiny tends to focus on structural and authority changes or outcomes. Politics and process of devolution implementation needs to be better understood to evaluate how national governments use the enhanced decision space for bringing improvements in the health system and the underlying challenges faced. We use the example of Pakistan’s radical, politically driven provincial devolution to analyse how national structures use decentralisation opportunities for improved health planning, spending and carrying out transformations to the health system. Our narrative draws on secondary data sources from the PRIMASYS study, supplemented with policy roundtable notes from Pakistan.

Our analysis shows that in decentralised Pakistan, health became prioritised for increased government resources and achieved good budgetary use, major strides were made contextualised sector-wide health planning and legislations, and a proliferation seen in governance measures to improve and regulate healthcare delivery. Despite a disadvantaged and abrupt start to devolution, high ownership by politicians and bureaucracy in provincial governments led to resourcing, planning and innovations. However, effective translation remained impeded by weak institutional capacity, feeble federal–provincial coordination and vulnerability to interference by local elites.

Building on this illustrative example, we propose (1) political management of decentralisation for effective national coordination, sustaining stable leadership and protecting from political interfere by local elites; (2) investment in stewardship capacity in the devolved structures as well as the central ministry to deliver on new roles.

**Summary box**

A process analysis of response to decentralisation in Pakistan and its consequences on health systems comes up with five main findings:

- Devolution led to increased government health allocations, sector-wide planning and governance innovations.
- Enthusiasm and ownership within subnational political–bureaucratic circles provided support.
- Weak national coordination capacity at centre and insufficient stewardship capacity in provinces impeded progress.
- Health systems became more vulnerable to local political interference requiring active management.
- Decentralisation implementation requires continued centre-province discussion on unresolved boundaries.

## Introduction

Decentralisation, in its various forms, is widely practised across low-income and middle-income countries (LMICs), including Kenya, Uganda, Nigeria and Ethiopia in Africa, Brazil, Peru and Mexico in Latin America, and Nepal, Indonesia and China in Asia.^1^ Technical arguments for decentralisation include local accountability, participatory governance, improved responsiveness and managerial efficiency.^2^ Administratively, decentralisation can be expressed in milder forms of delegating authority to a lower organisational level, in more advanced forms involving the transfer of authority to a lower or parallel administrative level, or devolution which is the total divesture of responsibility at the centre and its assumption by a lower tier.^3^

Scrutiny of decentralisation has predominantly focused on structural changes, extent of decision space provided or performance in terms of better equity, efficiency and accountability.^3–5^ We contend that decentralisation assessments are incomplete unless the process and politics of decentralisation is also examined. This is an important and relevant dimension as decentralisation is often brought in as part of a radical political change with demands for redistribution in resources, responsibilities and accountabilities,^6–9^ and performance is often unpredictable, mixed and dependent on the context in which decentralisation is implemented.^10^

In this paper, we present an analysis of improvements to health systems in Pakistan, in response to a politically driven devolution process. Devolution represents the most radical form of decentralisation. In 2010, Pakistan dissolved 17 federal ministries devolving legislative, operational and financial responsibilities to its four provinces.^11^ The devolution was undertaken through a major constitutional amendment unanimously supported by all political parties to meet the long-standing provincial demands for a lead role in shaping and implementing policies, a more equitable financial share and more locally responsive solutions particularly in the social sector.

The analysis aims to better understand and evaluate how subnational structures use decentralisation opportunities for improved planning, spending and carrying out transformations to the health system. Our narrative first landscapes the radical change in subnational authority and the process of abrupt devolution and subsequent partial recentralisation. Next, we analyse progress towards meeting devolution objectives in three areas: (1) did increased provincial resources translate into increased government expenditure for health?; (2) did enhanced provincial policy role lead to improvements in the health planning process?; (3) what progress was made in reform for health systems? Based on the above, we draw implications on how subnational governments used the enhanced decision space and influence of ownership, capacity, coordination and leadership factors.

The paper draw on findings from the Primary Care Systems Profile and Performance (PRIMASYS) Pakistan study conducted in 2016 by the authors. PRIMASYS probed structural aspects and processes of recent Primary Health Care planning, reforms and innovations.^12^ Additionally, minutes of national consultative policy round tables conducted for developing Pakistan’s National Health Vision 2016–25^13^ were analysed to identify success and barriers to post devolution health systems responses. This was supplemented with updates and insights from the authors (box 1).

Box 1 **Data sources: our narrative drew on the following secondary data sources:**

- Desk review conducted for PRIMASYS that included the National Finance Commission Award, post-devolution concurrent legislative list for health, provincial situation analysis reports, provincial health sector strategies, budgetary review of reconciled expenditure 2009/2010−2013/2014, provincial reform acts and legislations, organograms of national and provincial ministries, new PC-1s, important notifications, National Health Accounts, national health surveys, WHO mission reports, independent assessment reports.
- Analysis from 26 key informant interviews conducted during PRIMASYS: conducted to identify and explore two reform pathways—an initiative that progressed well and another that got hindered. Key informants were those who had been part of the post devolution initiative.
- Minutes of six national policy roundtables conducted for development of National Health Vision: 25–40 participants per roundtable, included representatives of national and provincial health ministries, NGOs, technical assistance agencies, experts, international development partners, private industry, medical and nursing associations.

## Devolution: extensive authority, abrupt process and partial recentralisation

Pakistan population of 207 million^14^ is spread over four provinces—Punjab, Sindh, Khyber Pakhtunkhwa and Baluchistan—and a small portion resides in federally controlled territories. Each province is geographically diverse, has a distinctive culture, regional language and distinct political following. Health has constitutionally been the provincial government’s responsibility; however, the presence of a concurrent legislative list allowed the fluid sharing of powers between the federal and provincial government.^15^ In practice, the federal ministry took lead in health planning, service delivery programming and monitoring, aid coordination, human resource and drug licensing and regulations. Over time, it also expanded into funding and management of the larger health hospitals.^16^ Provincial governments, while being a major co-financer of health, mainly had a passive role confined to administration of health facilities and programmes. Devolution was preceded by a radical change in federal–provincial resource distribution formula of 2009 with majority share (56%–58%) going to provinces.^17^ It is also an equity-based formula for distribution of resources to the less populated provinces by factoring in development needs and security challenges.^18^

Process of transitioning of power from federal to provincial government was abrupt. The 2010 devolution abolished the ‘concurrent legislative list’ and replaced it with an exclusive shorter list of federal powers and a longer list of exclusive provincial powers. The functions of health planning, legislation, service regulation, financing service delivery, human resource production and service delivery programming were devolved to the provinces (table 1). During the 14 months between devolution being promulgated into law (April 2010) to abolishment of the Ministry of Health (MoH) (June 2011), there was scant discussion and planning undertaken by the federal ministry with provinces. Hence, provinces were overnight confronted with additional responsibilities with resourcing and planning yet to be worked out.

**Table 1:** Distribution of federal–provincial roles and authority

Functions	Federal	Provincial
Health planning	International agreements and targets	Policies, strategies, plans, legislations
Financing	Co-financing preventive vertical programmes (interim arrangement) Insurance regulation	Financing curative+preventive Financing arrangements
Human resource	Licensing HR production	HR planning, deployment, management
Service delivery	Oversight on international agreements	Services menu, programming, implementation
Drug supply	Licensing, registration pricing	Market surveillance, supply systems
Health information system	Research Surveillance	Monitoring & Evaluation Surveillance
Governance	Standard setting	Strategic purchasing, regulation, accountability

Source: Federal Legislative List Parts I and II.

The provincial governments were not prepared for post devolution (budgetary) scenario. Only one workshop was conducted whereby future of National Institute of Health (NIH) Islamabad was discussed in light of devolution, but federal managed tertiary institutes were not discussed. As a result we had a fiasco at JPMC (Jinnah Postgraduate Medical Centre) and the matter went into litigation. (provincial health ministry)

The federal powers retained were dispersed across the remaining federal institutions such as the Planning Commission, Federal Bureau of Statistics^19^ and a newly set up Inter-Provincial Ministry. This posed issues in national coordination for global health commitments, drugs licensing, and regulation of medical and nursing professions. A multidonor WHO mission in 2012 recommended that dispersed federal powers be assembled into the Inter-Provincial Ministry for coherence.^20^ However, health functions were hurriedly reassembled into a separate federal ministry in 2013 during tenure of a caretaker government through a direct executive order, amalgamating a National Regualtions Ministry established in 2012 into the Ministry of National Health Services Regulation and Coordination (MoNHSRC). It has struggled to recover legitimacy as its existence is contested both by provinces as well as the federal entities to which its functions had originally been passed on. Post devolution, at least eight new federal ministries have been created without going through parliamentary approval process.^21^ Hence, the process of recentralisation started in the early years of devolution, but the remits of new central structures remains vague and unsettled.

## Government spending on health

Health spending post devolution depends largely on provincial contributions. A single line budgetary transfer is made to the provinces from the central tax revenue pool and then proportionate allocation to health and sectors by provinces in line respective provincial priorities. Provinces reacted to devolution through a steady and tangible increase in proportionate spending on health. Increased budget allocation for health is seen in all provinces ranging from 50% to threefold. Provincial contribution to the country’s government health expenditure has risen from 72% in 2009/10% to 82% in 2013/2014. There is also a visible rise in per capita health allocations by provincial governments (table 2). Increased provincial spending has also contributed to a faster growth of national consolidated government health expenditure in the country compared with the country’s overall health expenditure.^22^ Provincial budget execution rates have remained over 75% despite a sharp increase in health allocations.^22^ Strong ownership has been seen by the provincial political legislature in approving health budgets, constitution of provincial standing committees on health and prioritisation of health by chief ministers of all provinces.

**Table 2:** Health allocation and expenditure by provincial governments

	BE 2009/2010	BE 2010/2011	BE 2011/2012	BE 2012/2013	BE 2013/2014
Provincial per capita allocation on health (US$)					
Punjab	7.5	9.1	8.6	9.6	10.4
Sindh	7.0	7.9	8.8	10.9	12.9
Khyber Pakhtunkhwa	4.1	6.0	5.9	7.8	8.7
Baluchistan	6.2	9.0	11.3	11.9	15.6
Provincial health budget spending by salary vs non-salary (**%**)					
Punjab					
Salary	43%	48%	53%	57%	58%
Non-salary	57%	52%	47%	43%	42%
Sindh					
Salary	56%	58%	56%	55%	56%
Non-salary	44%	42%	44%	45%	44%
Khyber Pakhtunkhwa					
Salary	60%	68%	72%	68%	63%
Non-salary	40%	32%	28%	32%	37%
Baluchistan					
Salary	80%	66%	70%	73%	73%
Non-salary	20%	34%	30%	27%	27%

Source: consolidated annual health budgets of provincial finance departments; consolidated health expenditure from public accounts data 2009–2014; provincial population growth rates from National Census 2016–2017.

At the same time, there has been fiscal stress due to insufficient and delayed fiscal transfers from the health portion of federal budget that was committed pre-devolution. Federal health transfers comprise an important chunk of funding to financed vertical programmes, the extensive Lady Health Workers Program and tertiary hospitals under federal management. Insufficient federal fiscal transfers have precipitated doctors’ strikes at leading tertiary hospitals, protests by community health workers and stock out of supplies of vertical programmes.^23 24^ The gap has been partially adjusted by provinces through either own resources or international donor support.

Despite increased allocations, salaries continue to consume the major portion of the provincial health budgets and the share has even risen in some provinces (table 2). There is a policy push for recruitment of doctors and specialists rather than less costly frontline health workers. There is also a demand by provincial legislators for jobs provision to their constituents in government services.

## Stewardship and planning

In Pakistan, sector-wide planning was initiated for the first time after provincial devolution. Health policies were few and far between—only four health polices had been formulated in Pakistan’s 64-year history prior to devolution—these were mainly disease oriented, focused on the government sector delivery and not translated into operational planning.^16^ Planning had traditionally followed a project mode, shaped by specific government or donor-funded projects and vertical programmes. Project formulation was largely federally driven and had a rigidly tailored design across all provinces. Post devolution, provinces were confronted with a vacuum of sector-wide policy and planning for fulfilling the new stewardship function.

The devolution was thrown to the provinces with a single stroke of the pen; the provinces had neither the capacity nor were administratively ready to take this up. That is when we decided to develop provincial health sector strategy. (provincial health ministry)

Over the first 2 years of devolution, the provinces came up with province-specific Health Sector Strategies laying out a 10-year strategic direction across public and private health sectors. These were assisted by bilateral aid agencies and the World Bank, with donors viewing this as an opportune window to engage with provinces. In two of the provinces—Sindh and Khyber Pakhtunkhwa—the planning process moved forward to the district level with the development of District Health Plans. Roadmaps are in place in Punjab and Khyber Pakhtunkhwa to improve public service delivery using defined targets, regular stock-takes and investment in data. A significant increase in legislative activities has been seen across all four provinces. Legislations have for the first time been directed towards important health reform areas of Public Private Partnerships, Health Services Regulation and Autonomy of Teaching Hospitals. Certain level of restructuring of provincial Health Departments has also taken place to steer health stewardship. At least two provinces have established policy or reforms units, and one province has further restructured the health ministry to create a new department for primary and secondary care so as to ring fence administrative attention and resourcing for primary care (table 3).

**Table 3:** Provincial planning and governance initiatives

	Punjab	Sindh	Khyber Pukhtunkhwa	Baluchistan
Planning	Sector strategy developed. Roadmap for primary care in place	Sector strategy district action plans developed	Sector strategy and district action plans developed. Roadmap for primary care in place	Sector strategy developed
Regulatory authorities	Established and functional	Notified	Established and functional	Under consideration
Minimum service delivery package	Developed and costed	Developed and costed	Developed and costed	Developed and costed
Service delivery integration	Functional integration into three programmes	Functional integration proposed. Not implemented	Single integrated project, but parallel programmes coexist	Functional integration proposed. Not implemented
Private sector harnessing	New modalities: Regulatory health commission Contracting out of equipment/technology maintenance Contracting out of medicine and supplies delivery Contracting out of facilities maintenance	New modalities: Regulatory health commission Contracting-out management of secondary facilities in nine districts Contracting-out ambulance services, in selected districts	New modalities: Regulatory health commission Contracting-out started for district health systems in six districts, rolled back	Under consideration

However, health sector strategies, plans and legislations formulated remain unevenly implemented and only partially adjusted within health budgets.^19^ There are frequent instances of ad hoc initiatives for hospital infrastructure schemes, tertiary specialists units and medical colleges, popular with political leaderships and providing visibility for electorates. Capability is weak to build linkages between reform, planning and budgeting. Technical assistance for reforms provided by donors remains clustered in the better-performing resourced provinces of Punjab and Khyber Pukhtunkhwa, with less in Sindh and none in Baluchistan, hence further exacerbating inequities in terms of capability in terms of responding to devolution.

## *﻿*Governance innovations for improving primary healthcare

Post devolution, governance reforms to improve primary care service delivery have been attempted in all provinces but with varying success. We bring here two contrasting pathways taken to improve primary care delivery—the first example of private sector harnessing through contracting and regulation is an example that fared better whereas the second example of attempt towards integrating preventive healthcare delivery highlights failed resilience (table 3).

### Private sector harnessing

Pakistan has a mixed health system for primary care comprising a large network of government health facilities, private individual practitioners, philanthropic organisations and private medical entities. Government health facilities especially in rural areas have had low use, poor maintenance and poor quality of care.^25^ Post devolution, there are noticeable attempts to harness the private sector through regulation and purchasing of services.

Post devolution, government regulation of private providers is underway to reduce high levels of quackery and has been introduced in three provinces. There has also been a noticeable appetite among provinces to learn on private sector experiences from each other. Regulatory Health Commissions established in one province to regulate health services have been replicated in two other provinces. Licensing of facilities is well underway; however, the more ambitious quality assurance function lacks capacity. Legislative cover has been provided through the Public Private Partnerships and Health Services Regulation passed by the provincial assemblies.^26 27^ Another initiative to tighten market pharmacies and drug outlets is underway in one province, assisted by an expanded drug inspector workforce and computerised tagging of pharmacies.

Purchasing of private sector services has also gained policy foothold post devolution. The over-riding intent by provincial governments remains on improving the functionality of government health services through using private professionalised providers. Contracting the management of Basic Health Units (BHUs) NGO was first initiated in mid-2000s across all provinces by a federally led initiative,^28 29^ but based on single source contract. Post devolution, there has been (1) an expansion and diversification in number of private suppliers that include private hospital, development charities and private commercial firms; (2) expansion from BHU to contracting for rural health centres, district hospitals, referral hospitals and so on; (3) expansion from clinical to support services such as transportation of supplies, equipment maintenance and repairs, ambulance services and so on^30^ (table 3). Separate public–private partnership (PPP) units for contracting have been set up in the provincial health ministries of the two provinces for managing and monitoring PPPs, but there are serious capacity gaps in managing the contracting process to bring in qualified providers, link budgetary disbursements with outputs, and effectively monitor contracts.^31^

Provincial planners and implementers like to learn from each other and even compete in performing in certain areas such as regulation and public private partnerships. (independent expert)

### Integrating vertical healthcare programs

Pakistan has a long history of implementing vertical preventive health programmes, programmed by the federal ministry and co-financed by both federal and provincial ministries. At the time of devolution, there were 10 vertical programmes that include Expanded Program of Immunization, Roll Back Malaria, TB-DOTs, HIV AIDS Control, TB-DOTs, Maternal Newborn and Child Health, Nutrition Support Program, Blindness Control, Avian Flu, and Hepatitis B and C Control. Vertical programmes while providing the benefit of focused oversight have created fragmented service delivery, duplication of resources and delayed financial releases because of centralised budgets.^32–35^ Although vertical projects are required to be integrated into regular budgets and district staffing after a period of 4–5 years, some vertical programmes have been running for more than 30 years.

Post devolution, the declining federal support and challenge of expanding coverage to under-covered areas compelled provinces to re-look vertical programmes. Vertical programme integration was prioritised under provincial health sector strategies and costed Essential Health Service Delivery Packages were developed with donor technical assistance (table 3).^36–38^ One province moved ahead combining four overlapping programmes into a single integrated health project^39^ while another functionally integrated the 10 preventive programmes into three major programmes. Despite these beginnings, vertical programmes have largely continued in parallel. One of the blocks has been the continuation of federal funding line for vertical programmes and hampers creation of integrated budgeting. A fresh cycle of vertical projects has been started by the new federal health ministry and posts of federal vertical programme directors have been revived, hence re-establishing federal verticalised authority over preventive care. Constraints for integration are also seen in the provinces. Vertical programme integration is actively resisted by provincial vertical programme managers, who often enjoy backing from provincial legislatures—this has moved integration from a high-priority agenda to a de-prioritised agenda.

Behind every vertical program is a politician. How can change be brought in such a context? (vertical programme manager)

### Leadership support, capacity and federal–provincial coordination

Proliferation in health systems initiatives post devolution has been driven by strong support by provincial governments and the civil bureaucracy. At the same time, key factors need to be understood that have constrained effective translation.

The provinces had a disadvantaged start in terms of technical capability as the past context of federally dominated political, fiscal, administrative power stunted the maturity of provincial administrative structures. Strong political support for devolution and visible ownership of health in all four provinces led to increased resourcing of health from government budget, push for provincial planning and monitoring, as well as support for regulation, contracting and other governance initiatives to circumvent slow-moving government systems to deliver results. At the same time, the loosening of federal verticality made the provinces vulnerable to interference by provincial political elites. Political pressure for visible health infrastructure projects, specialty schemes, recruitment of local constituents and instances of favoured appointments was seen in all provinces, often derailing health sector strategies. Provinces where health ministries had strong executive backing were better able to withstand political pressures than those that were not closely aligned to provincial leadership. Trained human resource and lack of support systems has blunted progress of sector strategies despite an appetite for health reforms. Frequent change in health secretaries in all provinces further interrupted the momentum of progress.

In Provinces A&B __ there is clear divide in (provincial) government on ethnic grounds. This has resulted in divided bureaucracy and (politically) always a coalition government with ministers maneuvering their own chosen Secretaries and other staff in clear violation of merit and seniority. This results in a kind of institutional and governance break down. (provincial planning and development department)

Frequent transfer and postings at senior management level especially of Secretary of Health results in coordination gaps to address health issues and also a lack of interest at all levels. (development partner).

Weak federal–provincial coordination is also responsible for slowing transformative change. Transition of devolutionary powers was abrupt taking place without planning discussions between the federal ministry with provincial counterparts. As a result, there continue to be unresolved resourcing and administrative issues, dodging the federal–provincial relationship over the past 7 years. Federal dialogue with provinces has remained ad hoc post devolution and is usually precipitated by crisis such as polio outbreaks or vertical initiatives championed by MoNHSRC. As yet, there has been a lack of national leadership to address outstanding federal–provincial issues, coordinate on a common national direction and allow lessons sharing across provinces.

The federal ministry also has capacity issues—it has been re-populated with former staff experienced in vertical programme management, but lacks expertise for its new role of coordination and regulation across the diverse provinces.^19^ Technical assistance provision to the federal health ministry has been overlooked by international donors and placed solely to the sources. Above all, the ministry lacks federal political clout, perceived in executive circles as a low resourced ministry with ill-defined functions.

## Conclusion

We assessed Pakistan’s 2010 provincial health devolution against devolution’s intended outcomes of better resourcing, improved planning and local contextual innovations. By focusing on the critically less understood area of processes and politics, we explored why certain initiatives progressed better and other did not, so as to better manage decentralisation experiences. Our analysis did not assess performance outcomes and its attribution to specific health reforms post devolution.

In decentralised Pakistan, health became prioritised for increased government resources and achieved good budgetary use. However, allocation priorities were not effectively adjusted and there was little effort at targeting of inequities. Major strides were made in terms of provincially contextualised sector-wide health planning and legislations, but implementation has been weakly steered. There has also been a feverish proliferation in governance measures to improve, professionalise and regulate healthcare delivery. Some have had wide roll-out, others have been only partially implemented and at times, even aborted.

Global findings show that political decentralisation is associated with higher health expenditures and even higher in case of both political and fiscal decentralisation.^40–43^ Latin American countries’ experience shows decentralised governments were successful in mobilising additional household sources,^8 44^ which we did not see in the case of Pakistan. Decentralisation experiences in Kenya,^45 46^ Nigeria^47^ and Mexico^48^ report weak mechanisms for resource management in subnational governments. There is scant evidence from developing economies on post devolution planning and governance; however, literature from Sweden and UK supports the case of improved regional planning and innovations post decentralisation.

Our analysis shows that high political-bureaucratic ownership of health facilitated progress but weak stewardship skills and frequent leadership changes at subnational level, vulnerability to interference by local elites and feeble national coordination constrained effective implementation. The federal–provincial relationship post devolution remained troublesome having a disadvantaged start with abrupt transitioning, unresolved resource sharing issues and ad hoc vertically led dialogue. There is scant literature on politics of health decentralisation in LMICs. Chaotic interprovincial coordination is reported from West and Central African states, where the central government resisted relinquishing resources and attempted to re-empower the central government.^44 49–53^ LAC experience analysed by Bossert *et al* highlights the value of incentives to make subnational structures perform which was missing in Pakistan’s decentralisation design government.

We contend that examination of decentralisation should be expanded to include process assessments to detect challenges and help manage responses. We propose a few recommendations. First, investment for technical capability development in devolved structures is required early on as most decentralisation experiences in LMICs, driven by sweeping political process, will not provide time for a learning-by-doing incrementalist approach seen in technical driven experiments with decentralisation in OECD countries. Second, technical support for the central level to shift to a new modus operandi should not be over-looked as part of capacity building efforts. Third, measures are needed for political management of decentralisation to safeguard against local political pressures, leadership stability, and continued centre-province discussion on issue interpretation and consensus building.
